# Stimulation of neutrophil functions by C5a_desArg_: an in vitro model of haemodialysis

**DOI:** 10.1155/S0962935192000127

**Published:** 1992

**Authors:** I. Eckle, G. Kolb, E. Martinez Martin, K. Havemann

**Affiliations:** 1 Centre of Internal Medicine Philipps-University Baldinger Strasse Marburg D-355 Germany

## Abstract

Cuprophane membranes during haemodialysis significantly increase the plasma levels of C5a_desArg_ (maximal 55 μg C5a_adesArg_/1 blood after 30 min) whereas Hemophane or Polysulphonemembranes induce only low plasma levels of C5a_desArg_. C5a_desArg_ generated in vitro by yeast incubation of autologous plasma stimulates PMN chemotaxis and oxidative metabolism but has no effect on enzyme release. Preincubation of whole blood with C5a_desArg_ causes aggregation and changed oxidative burst activity of the isolated PMN. These changes are similar to those found in cells from patients after haemodialysis with cuprophane membranes. So the elevated plasma levels of C5a_desArg_ after haemodialysis explain some of the changes in PMN functions, but additional mechanisms have to be assumed.


Mediators of Inflammation 1, 61–66 (1992)

### Stimulation of neutrophil functions by $C5a_{desArg}$: an *in vitro* model of haemodialysis

I. Eckle[CA], G. Kolb, E. Martinez Martin and K. Havemann

Centre of Internal Medicine, Philipps-University, Baldinger Strasse, D-355 Marburg, Germany

[CA] Corresponding Author

**Cuprophane membranes during haemodialysis significantly increase the plasma levels of $C5a_{desArg}$ (maximal 55 μg $C5a_{desArg}$/l blood after 30 min) whereas Hemophane or Polysulphonemembranes induce only low plasma levels of $C5a_{desArg}$. $C5a_{desArg}$ generated *in vitro* by yeast incubation of autologous plasma stimulates PMN chemotaxis and oxidative metabolism but has no effect on enzyme release. Preincubation of whole blood with $C5a_{desArg}$ causes aggregation and changed oxidative burst activity of the isolated PMN. These changes are similar to those found in cells from patients after haemodialysis with cuprophane membranes. So the elevated plasma levels of $C5a_{desArg}$ after haemodialysis explain some of the changes in PMN functions, but additional mechanisms have to be assumed.**

**Key words:** $C5a_{desArg}$, Cuprophane, Haemodialysis, PMN functions

### Introduction

During haemodialysis (HD) with various membranes complement components are activated, which results in elevated levels of C5a and C3a.[1,2] Additionally neutrophil granulocytes (PMN) are activated, which results in the release of lysosomal enzymes[3] and an altered oxidative metabolism.[4] To explain the change in the PMN functions two effects have to be evaluated: a direct effect of the membrane material on PMN and an indirect effect of membrane activated plasma components on the cells. If the two effects are exerted, they may differ in their extent, in their kinetic, and in their dependence on the membrane material. They can be studied in two ways: by using membrane activated cells without the addition of plasma[5] and by using normal cells with activated plasma.[1]

In this study we set out to investigate the incubation of normal PMN with yeast activated plasma as an *in vitro* model of the plasma effects during haemodialysis.

### Material and Methods

*Isolation of human PMN:* Blood was drawn by venipuncture after obtaining written consent from normal, healthy donors. PMN were isolated using Ficoll (Pharmacia-LKB, Bromma, Sweden) separation and sedimentation of erythrocytes.[6] The cells represented more than 98% granulocytes with a viability of about 98% by Trypan exclusion.

*Generation of $C5a_{desArg}$:* Autologous plasma was activated by incubation with baker's yeast (1 mg $ml^{-1}$) for 60 min at 37°C. Plasma was inactivated by incubation for 30 min at 56°C.

Concentration of C5a and $C5a_{desArg}$ was measured with an ELISA (Behringwerke, Marburg, Germany).

*Chemotaxis assay:* Chemotactic assays were performed in modified Boyden chambers.[7] Activated plasma (2.5–20%) in HBSS (Gibco, Germany) was put in the lower compartment and the PMN ($10^6$) in the upper compartment of the Boyden chambers. Cellulose ester filters (ME 29, Schleicher and Schüll, Dassel, Germany) were used. The chambers were incubated for 90 min at 37°C, then the filters were removed, stained with hematoxylin, and mounted on slides. The PMN were counted in the filters at every 10 μm interval by a computerized imaging method (Bausch and Lomb, Frankfurt, Germany) and the chemotactic index (CI) calculated, which reflects the mean distance travelled by the activated cells.[7]

*Cytochrome c test:* Superoxide anion generation was measured by cytochrome c reduction.[8] PMN ($10^6$) were incubated with 75 μM cytochrome c and stimuli (Phorbol 12-myristate 13-acetate, PMN, 1 μg $ml^{-1}$ or activated plasma 0.3–20% as indicated in results) in Hanks' balanced salt solution (HBSS) for 10 min at 37°C. The reaction was stopped by cooling in ice and centrifugation (10 min, 200 × *g*). The absorption of the supernatants was determined at 550 nm, and the superoxide generation was calculated.[9]

*Elastase release:* PMN (4 × $10^6$) were incubated with activated plasma (2.5–20% as indicated in results) in HBSS for 15 min at 37°C. Released elastase (HLE) was complexed with a1-proteinase inhibitor (a1PI) by addition of plasma (25%) and further

© 1992 Rapid Communications of Oxford Ltd incubated for 2 min. The samples were centrifuged (10 min, 200 × *g*) and the HLE-a1PI-complex was measured by ELISA (Merck, Darmstadt, Germany). Total HLE content of the PMN was measured after lysis with cetyltrimethylammonium bromide.[10]

*Preincubation experiments:* Sixty ml of anticoagulated blood were centrifuged and the plasma separated. Ten ml of the plasma were activated and 10 ml inactivated as described above. The blood was then divided into three parts and each was treated in one of the following ways: (1) PMN were directly isolated from one part, (2) one part of the blood was mixed with activated plasma (final concentration 10%) and incubated for 10 min at 37°C, then the PMN were isolated, (3) one part of the blood was mixed with inactivated plasma (final concentration 10%) and treated as part 2.

*Patient studies:* Twenty-seven patients gave their informed consent to participate in the study. Haemodialysis (HD) was performed by an A 2008 C (Fresenius, Bad Homburg, Germany) or an AK-10 (Gambro, Hechingen, Germany) volume controlled equipment. As our dialyser we used cuprophane: SMAD 125/140 (SMAD, Lyon, France), hemophane: MO 450 (SMAD) and GFS 120/140 MCH (Gambro, Lund, Sweden), and polysulfone: F6 (Fresenius).

EDTA blood was taken from the arterial line before systemic heparinization prior to HD and 10, 30, 60, 120 and 180 min after the beginning of HD. The blood was immediately centrifuged and the plasma stored deep frozen until assessed for C5a by ELISA.

Chemicals were if not otherwise stated by Sigma Chemie, Deisenhofen, Germany.

*Statistical analysis:* Data are represented as the mean ± 1 standard deviation (SD). Statistical comparisons were performed by the paired Student's *t*-test.

### Results

*$C5a_{desArg}$ generation during dialysis:* Figure 1 shows the kinetics of $C5a_{desArg}$ generation in the venous blood samples during haemodialysis (HD). HD with Cuprophane gives maximal levels of 55 μg $C5a_{desArg}$/l after 30 min. HD with Hemophane Mo 450 or GF 120 results in lower values of maximal 8 μg $C5a_{desArg}$/l after 10 min of dialysis. Similar results are obtained for HD with Polysulfone (maximal 5 μg $l^{-1}$).

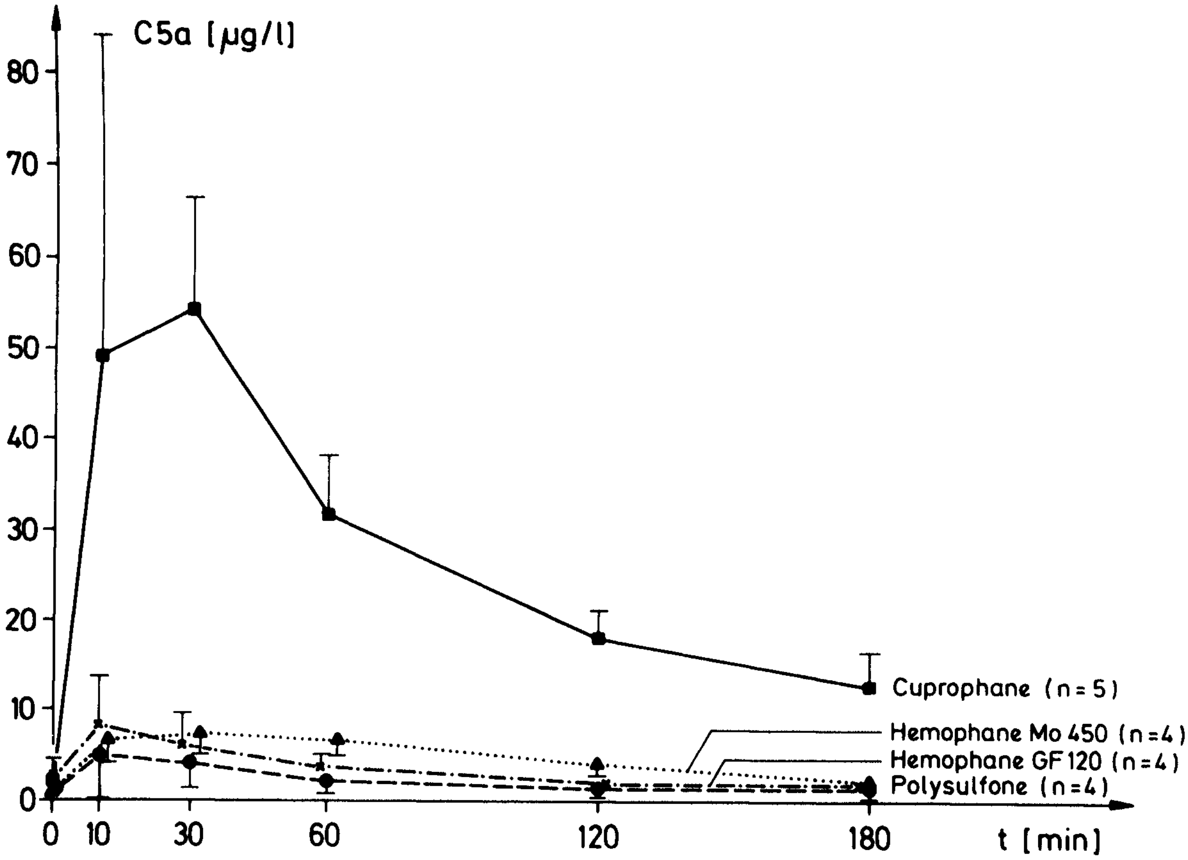

FIG. 1. Change of $C5a_{desArg}$ concentration as a function of dialysis time for different membrane materials.

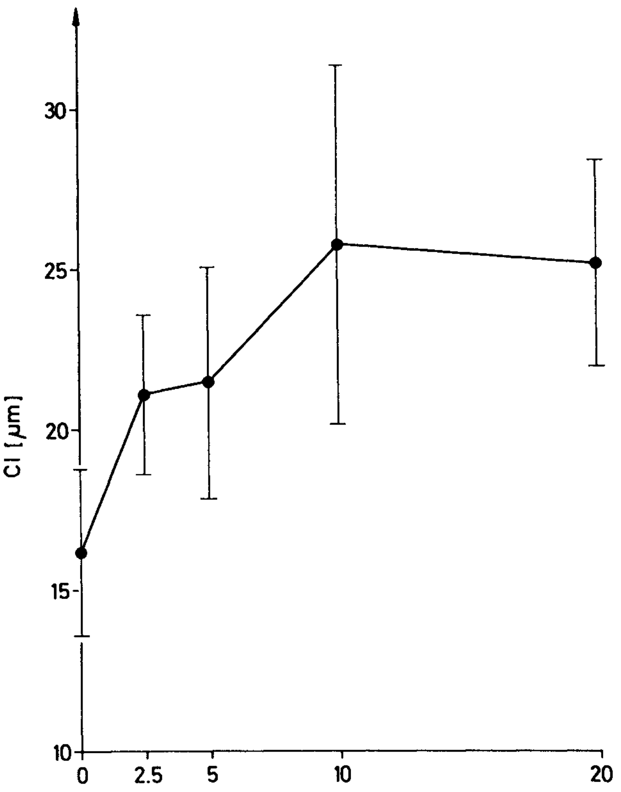

FIG. 2. Chemotaxis of human PMN stimulated with activated plasma. Autologous plasma was activated and diluted in HBSS (final concentration given on the abscissa). The cell migration was assayed for 90 min at 37°. Data represent the mean ± SD of five separate experiments done in duplicate.

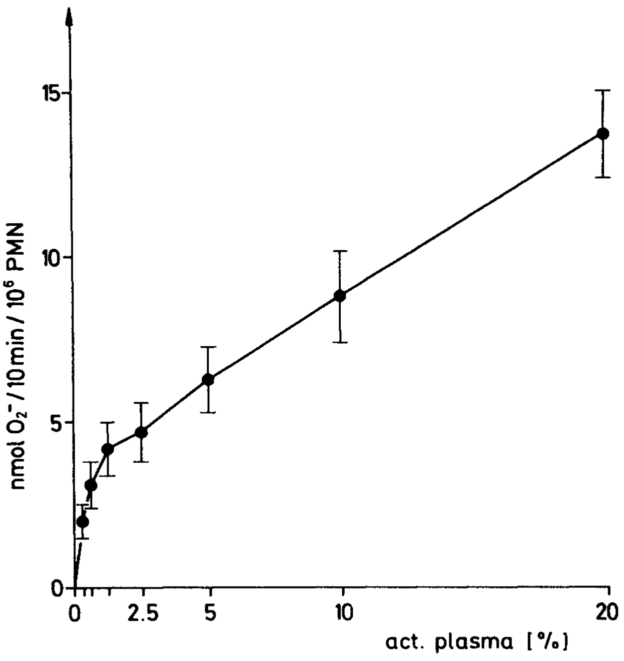

FIG. 3. Superoxide anion generation of human PMN stimulated with activated plasma. Autologous plasma was activated and incubated (in the final concentration given on the abscissa) with PMN ($10^6$) and cytochrome c (75 μM) for 10 min at 37°C. Data represent the mean ± SE of three separate experiments done in duplicate.

*In vitro generation of C5a$_{desArg}$*: Generation of C5a$_{desArg}$ by yeast activation of autologous plasma samples results in a mean value of 1040 ± 450 μg C5a$_{desArg}$/l ($n = 8$). In parallel inactivated plasma samples contain 1.7 ± 0.9 μg C5a$_{desArg}$/l ($n = 8$).

*Stimulation of chemotaxis by activated plasma:* Figure 2 shows the concentration dependent stimulation of chemotaxis by the autologous plasma samples. Maximal stimulation is achieved by 10% activated plasma which corresponds to about 100 μg C5a$_{desArg}$/l.

*Stimulation of oxidative burst by activated plasma:*

*Direct effect of activated plasma.* The superoxide generation of PMN is already stimulated by 1% of activated plasma corresponding to about 10 μg C5a$_{desArg}$/l to 2 nmol $O_2^-$/10 min/$10^6$ PMN (Fig. 3). Further addition of activated plasma up to 20% (about 200 μg C5a$_{desArg}$/l) enhances the superoxide production to 13.7 nmol/10 min/$10^6$ PMN.

*Preincubation of PMN with activated plasma.* To test the effect of priming, blood was mixed with activated or inactivated plasma (final concentration 10%) and incubated for 10 min at 37°C, and then the PMN were isolated. As a control, PMN from the same donor were directly isolated.

The yield of PMN is reduced to 18% of the control after incubation with activated plasma (Table 1, $p < 0.002$). Incubation of the blood with inactivated plasma also reduces the yield (55% of control, not significant = NS). If the superoxide generation of the isolated PMN is tested without the addition of a stimulus, the plasma preincubated cells produce higher amounts of superoxide than untreated cells (4.5 and 4.1 vs. 2.2 nmol $O_2^-$/$10^6$ PMN/10 min, NS, Fig. 4). In contrast, generation of superoxide is significantly reduced after PMN stimulation of the C5a$_{desArg}$ preincubated cells: 9.5 nmol $O_2^-$/10 min/$10^6$ PMN vs. 20.1 nmol of control cells ($p < 0.02$) and 17.5 nmol of cells incubated with inactivated plasma ($p < 0.01$). After stimulation with activated plasma the C5a$_{desArg}$ pretreated cells produce more superoxide than the other groups: 9.3 nmol/10 min/$10^6$ PMN vs. 5.5 nmol of control cells (NS) and 5.1 nmol of inactivated plasma incubated cells ($p < 0.05$).

**Table 1.** Yield of PMN after preincubation of the blood with activated or inactivated plasma (final concentration 10%, 10 min, 37°C) compared to untreated control blood

| Preincubation | PMN/20 ml blood | Yield (%) |
|---|---|---|
| control | $3.8 \pm 2.0 \times 10^7$ | 100 |
| 10% act. plasma | $6.8 \pm 5.2 \times 10^6$ | 18 |
| 10% inact. plasma | $2.1 \pm 1.6 \times 10^7$ | 55 |

Statistical significance by Student's *t*-test: act.plasma vs. control $p < 0.002$, act. vs. inact. plasma $p < 0.05$.

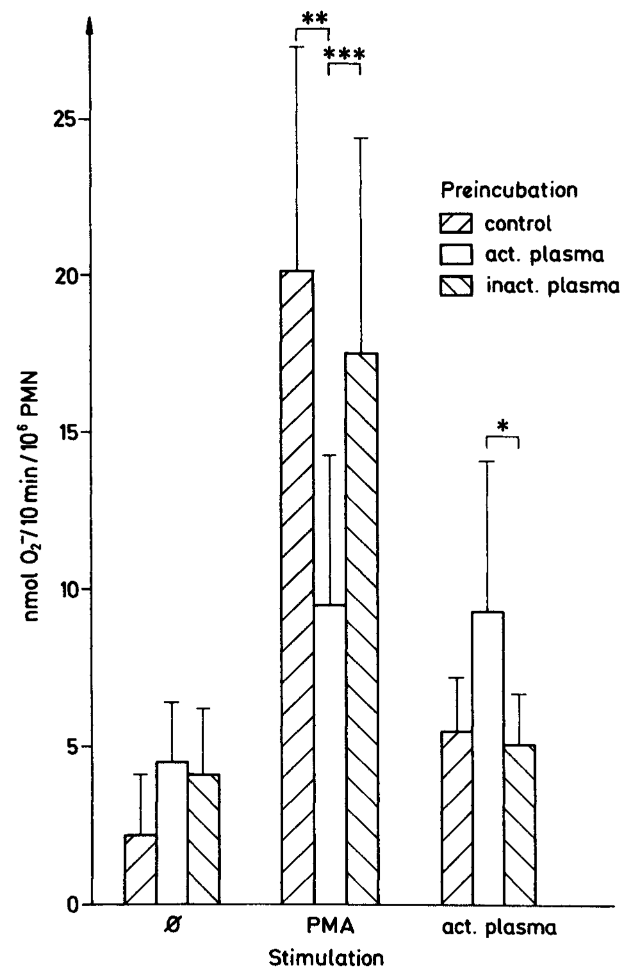

FIG. 4. Superoxide anion generation of human PMN after preincubation with autologous plasma. PMN were directly isolated from blood (control) or the blood was incubated (37°C, 15 min) either with 10% activated plasma (act. plasma) or with 10% inactivated plasma (inact. plasma) before isolation. Superoxide anion generation was measured for 10 min at 37°C without addition of stimulus (0), after stimulation with PMA (1 $\mu g\ ml^{-1}$) (PMA) or with activated plasma (final concentration 20%) (act. plasma). Data represent the mean $\pm$ SD of eight separate experiments done in duplicate. Statistical significance by Student's *t*-test: $*p < 0.05$, $**p < 0.02$, $***p < 0.01$.

Preincubation of blood with lower concentrations of activated plasma (final concentration 5%) gives similar results as preincubation with inactivated plasma (data not shown).

*Stimulation of HLE release:* Incubation of isolated PMN with activated plasma results in no stimulation of HLE release in the range of 1–20% activated plasma.

### Discussion

Human C5a is cleaved from the fifth component of complement during activation and then rapidly converted by a plasma enzyme to the $C5a_{desArg}$ derivative.[11] The plasma levels of $C5a_{desArg}$ in healthy adults are normally below 0.2 $\mu g\ l^{-1}$.[12]

The measurement of C5a or $C5a_{desArg}$ is difficult because of its short half-life.[13,14] Measurements

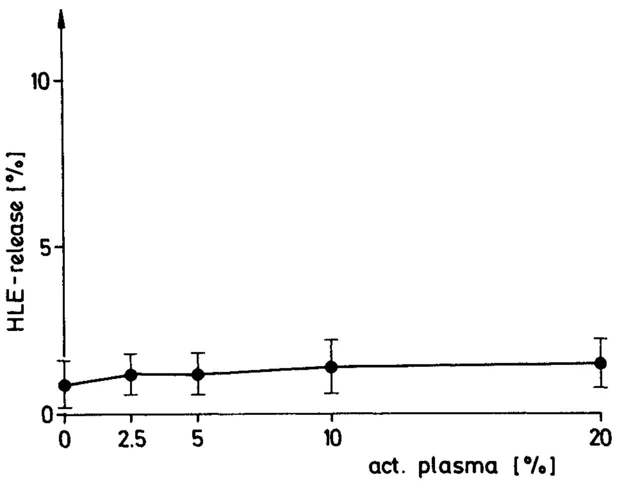

FIG. 5. HLE release of human PMN stimulated with activated plasma. Autologous plasma was activated and incubated with PMN ($4 \times 10^6$) in the final concentration given on the abscissa for 15 min at 37°C. Data represent the mean $\pm$ SD of six separate experiments done in duplicate.

during haemodialysis have to be made in venous blood samples which come directly from the dialyser, where C5a is supposed to be generated.[1,15]

We found maximal plasma levels of 55 $\mu g$ $C5a_{desArg}$ after 30 min of haemodialysis with cuprophan membranes (Fig. 1) in accordance with results of Knudsen, Bingel and Freyria[16–18] (maximal 35–61 $\mu g\ l^{-1}$). The other membranes only produced about 8 $\mu g$ $C5a_{desArg}$ (Fig. 1).

For our *in vitro* experiments we generated $C5a_{desArg}$ by yeast activation of the autologous plasma samples, which resulted in 1040 $\pm$ 450 $\mu g$ $C5a_{desArg}/l$ on average, while the inactivated samples only contained about 2 $\mu g\ l^{-1}$. To test the $C5a_{desArg}$ activity we performed chemotaxis experiments (Fig. 2). Maximal stimulation was achieved by 10% activated plasma corresponding to 60 nM $C5a_{desArg}$, the same value as described by Yancey *et al.*[19] and Chenoweth and Hugli.[20] The native C5a fragment was about 20-fold more potent ($EC_{50}$ C5a 0.5 nM).[21]

The stimulation of the oxidative burst with activated plasma (Fig. 3) gave no maximum but was increased in the whole range tested (final concentration 1–20% activated plasma) as already described by Goldstein.[22]

So we found stimulation of chemotaxis and oxidative burst by about 10% activated plasma, which corresponded to 100 $\mu g$ $C5a_{desArg}/l$, the same values found after haemodialysis with cuprophan membranes. To simulate the complement exposure during dialysis, we preincubated blood with 10% activated plasma (final concentration 100 $\mu g$ $C5a_{desArg}/l$ blood) and as the control with 10% inactivated plasma (final concentration 0.2 $\mu g$ $C5a_{desArg}/l$), and tested the subsequently isolated PMN.

$C5a_{desArg}$ aggregates PMN[23,24] and the aggregated cells were separated by Ficoll centrifugation. So the yield of PMN was rather reduced after incubation with activated plasma (Table 1). The prestimulated PMN produced less superoxide after further stimulation with PMA than the control cells or the cells that were preincubated with inactivated plasma (Fig. 4). The extent of these effects differed from one blood donor to the other, reflected by the large standard deviations, but the ratio of the values was always the same.

So the preincubation experiments imitate the *in vivo* results, which also show leukopenia after cuprophan dialysis[25,26] and diminished superoxide generation of the isolated PMN.[4]

It seems probable that after stimulation a subpopulation of PMN with lower activity was isolated,[27,28] because priming of PMN with a stimulus normally resulted in enhanced activity towards a second stimulus.[29] However, if the prestimulated PMN are further stimulated with activated plasma, the superoxide generation is enhanced in comparison to untreated cells (Fig. 4). So the activated plasma seems to contain a factor which compensates the cellular effect. These apparently contradictory results are also produced during dialysis, where the superoxide generation of PMN in autologous plasma is enhanced at the same time as the superoxide generation of isolated PMN is reduced.[30,31]

Our preincubation experiments were performed with high $C5a_{desArg}$ concentrations which correspond to the values after cuprophane dialysis. And they show similar results for PMN function as are produced during cuprophane dialysis. Preincubation with lower $C5a_{desArg}$ concentrations (5% activated plasma corresponding to a final concentration of about 50 $\mu$g $C5a_{desArg}$/l) shows no effect. But other membrane materials such as hemophane also induce leukopenia and changes of PMN oxidative burst although they only generate very small amounts of $C5a_{desArg}$.[4,16–18] So $C5a_{desArg}$ may be responsible for the complement induced effects after cuprophan dialysis but not for the effects after HD with the other membranes. This is confirmed by several other authors who find no correlation between the extent of complement activation and leukopenia.[32–35] Moreover complement activation by cuprophane also depends on additional serum factors which differ greatly between individuals.[36]

The second cellular effect during dialysis, the enzyme release,[37,4] is not mimicked by activated plasma. Incubation of isolated PMN with up to 20% activated plasma results in no release of HLE (Fig. 5) Release of azurophil granules is only stimulated after cytochalasin b preincubation[38] while only specific granules are released by soluble stimuli.[39] So the HLE release of PMN during dialysis[40] can not be explained by the activated plasma components but additional stimulating effects of the dialysis membrane have to be assumed.

$C5a_{desArg}$ generated during haemodialysis may influence some of PMN functions e.g. the oxidative burst. But for other functions e.g. the enzyme release an additional influence of the membrane material has to take effect.

### References

1. Craddock PR, Fehr J, Dalmasso AP, *et al.* Haemodialysis leukopenia: pulmonary vascular leukostasis resulting from complement activation by dialyser cellophane membrane. *J Clin Invest* 1977; **59:** 879–888.
2. Chenoweth DE. Complement activation during haemodialysis: clinical observations, proposed mechanisms and theoretical implications. *Int J Artif Organs* 1984; **8:** 281–287.
3. Hörl WH, Feinstein EI, Wanner C, *et al.* Plasma levels of main granulocyte components during haemodialysis. *Am J Nephrol* 1990; **10:** 53–57.
4. Kolb G, Fischer W, Müller T, *et al.* Granulocyte-related bioincompatibility of haemodialysis: inhibition of oxidative metabolism, degranulation reaction, enzyme release and leukocyte sequestration in the lung. *Int J Artif Organs* 1989; **12:** 294–298.
5. Kolb G, Nolting C, Eckle I, *et al.* The role of membrane contact in haemodialysis-induced granulocyte activation. *Nephron* 1991; **57:** 64–68.
6. Böyum A. IV. Isolation of mononuclear cells and granulocytes from blood—isolation of mononuclear cells by one centrifugation and of granulocytes by combining centrifugation and sedimentation of 1g. *Scand J Clin Lab Invest* 1967; **21(Suppl 97):** 77–89.
7. Maderazo EG, Woronick CL. Micropore filter assay of human granulocyte locomotion: problems and solutions. *Clin Immunol Immunopathol* 1978; **11:** 196–201.
8. Babior BM, Kipnes RS, Curnutte JT. Biological defence mechanisms. The production of leukocytes of superoxide, a potential bactericidal agent. *J Clin Invest* 1973; **52:** 741–744.
9. Massey V. The microestimation of succinate and the extinction coefficient of cytochrome c. *Biochem Biophys Acta* 1959; **34:** 255–257.
10. Ganz T. Extracellular release of antimicrobial defensins by human polymorphonuclear leukocytes. *Infect Immun* 1987; **55:** 568–571.
11. Hugli TE. The structural basis for anaphylatoxin and chemotactic functions of C3a, C4a, and C5a. *CRC Crit Rev Immun* 1981; **1:** 321–366.
12. Klos A, Ihring V, Messner M, *et al.* Detection of native human complement components C3 and C5 and their primary activation peptides C3a and C5a (anaphylatoxic peptides) by ELISAs with monoclonal antibodies. *J Immunol Meth* 1988; **111:** 241–252.
13. Webster RO, Larsen GL, Henson PM. *In vivo* clearance and tissue distribution of C5a and C5a des Arginine complement fragments in rabbits. *J Clin Invest* 1982; **70:** 1177–1183.
14. Weisdorf DJ, Hammerschmidt DE, Jakob HS, *et al.* Rapid *in vivo* clearance of $C5a_{desArg}$, a possible protective mechanism against complement-mediated tissue injury. *J Lab Clin Med* 1981; **98:** 823–830.
15. Chenoweth DE, Cheung AK, Henderson LW. Anaphylatoxin formation during haemodialysis: effects of different dialyser membranes. *Kidney Int* 1983; **24:** 764–769.
16. Knudsen F, Nielsen AH, Pedersen JO, *et al.* On the kinetics of complement activation, leucopenia and granulocyte-elastase release induced by haemodialysis. *Scand J Clin Lab Invest* 1985; **45:** 759–766.
17. Bingel M, Arndt W, Schulze M, *et al.* Comparative study of C5a plasma levels with different haemodialysis membranes using an enzyme-linked immunosorbent assay. *Nephron* 1989; **51:** 320–324.
18. Freyria AM, Leitienne Ph, Veysseyre CN, *et al.* Complement C3 and C5 degradation products during haemodialysis treatment: study of an index of membrane bioincompatibility. *Int J Artif Organs* 1988; **11:** 111–118.
19. Yancey KB, Lawley TJ, Dersookian M, *et al.* Analysis of the interaction of human C5a and $C5a_{desArg}$ with human monocytes and neutrophils: flow cytometric and chemotaxis studies. *J Clin Dermatol* 1989; **92:** 184–189.
20. Chenoweth DE, Hugli TE. Human C5a and C5a analogs as probes of the neutrophil C5a receptor. *Mol Immunol* 1980; **17:** 151–161.
21. Psychoyos S, Uziel-Fusi S. Comparison of LTB4- and C5a-stimulated chemotaxis of isolated human neutrophils: difference revealed by cell migration in thick filters using the multiwell cap procedure. *Agents & Actions* 1989; **27:** 380–384.
22. Goldstein IM, Roos D, Weissmann G, *et al.* Complement and Immunoglobulins stimulate superoxide production by human leukocytes independently of phagocytosis. *J Clin Invest* 1975; **56:** 1155–1163.
23. Hammerschmidt DE, Bowers TK, Lammi-Keefe CJ, *et al.* Granulocyte aggregometry: A sensitive technique for the detection of C5a and complement activation. *Blood* 1980; **55:** 898–902.
24. Craddock PR, Hammerschmidt D, White JG, *et al.* Complement (C5a)-induced granulocyte aggregation *in vitro*. *J Clin Invest* 1977; **60:** 260–264.
25. Dodd NJ, Gordge MP, Tarrant J, *et al.* A demonstration of neutrophil accumulation in the pulmonary vasculature during haemodialysis. *Proc EDTA* 1983; **20:** 186–189.

26. Kolb G, Höffken H, Müller T, *et al.* Kinetics of pulmonary leukocyte sequestration in man during haemodialysis with different membrane-types. *Int J Artif Organs* 1990; **11**: 729–736.
27. Klempner MS, Gallin JI, Balow JE, *et al.* The effect of haemodialysis and $C5a_{desArg}$ on neutrophil subpopulations. *Blood* 1980; **55**: 777–783.
28. Cohen MS, Elliott DM, Chaplinski T, *et al.* A defect on the oxidative metabolism of human polymorphonuclear leukocytes that remain in circulation early in haemodialysis. *Blood* 1982; **60**: 1283–1288.
29. Van Epps DE, Garcia ML. Enhancement of neutrophil function as a result of prior exposure to chemotactic factor. *J Clin Invest* 1980; **66**: 167–175.
30. Markert M, Heierli C, Kuwahara T, *et al.* Dialysed polymorphonuclear neutrophil oxidative metabolism during dialysis: a comparative study with five new and reused membranes. *Clin Nephrol* 1988; **29**: 129–136.
31. Nguyen AT, Lethias C, Zingraff J, *et al.* Haemodialysis membrane-induced activation of phagocyte oxidative metabolism detected *in vivo* and *in vitro* within microamounts of whole blood. *Kidney Int* 1985; **28**: 158–167.
32. De Vinuesa SG, Resano M, Luno J, *et al.* Leucopenia, hypoxia and complement activation in haemodialysis. Three unrelated phenomena. *Proc EDTA* 1982; **19**: 159–167.
33. Danielson BG, Hällgren R, Venge P. Neutrophil and eosinophil degranulation by haemodialysis membranes. *Contr Nephrol* 1984; **37**: 83–88.
34. Heierli C, Markert M, Lambert PH, *et al.* On the mechanism of haemodialysis-induced neutropenia: a study with five new and re-used membranes. *Nephrol Dial Transplant* 1988; **3**: 773–783.
35. Hörl WH, Riegel W, Schollmeyer P, *et al.* Different complement and granulocyte activation in patients dialysed with PMMA dialysers. *Clin Nephrol* 1986; **25**: 304–307.
36. Maillet F, Kazatchkine MD. Specific antibodies enhance alternative complement pathway activation by cuprophane. *Nephrol Dial Transplant* 1991; **6**: 193–197.
37. Hörl WH, Schäfer RM, Heidland A. Effect of different dialysers on proteinases and proteinase inhibitors during haemodialysis. *Am J Nephrol* 1985; **5**: 320–326.
38. Fantozzi R, Brunelleschi S, Soldaini GB, *et al.* *N*-formylmethionyl-leucyl-phenylalanine: different releasing effects on human neutrophils and rat mast cells. *Agents & Actions* 1983; **13**: 218–221.
39. Zimmerli W, Reber A-M, Dahinden CA. The role of formylpeptide receptors, C5a receptors, and cytosolic-free calcium in neutrophil priming. *J Infect Dis* 1990; **161**: 242–249.
40. Schäfer RM, Herfs N, Ormanns W, *et al.* Change of elastase and cathepsin G content in polymorphonuclear leukocytes during haemodialysis. *Clin Nephrol* 1988; **29**: 307–311.

**Received 18 October 1991; accepted in revised form 13 December 1991**

## References

[PDF_00417] Babior B. M., Kipnes R. S., Curnutte J. T. (1973). Biological defense mechanisms. The production by leukocytes of superoxide, a potential bactericidal agent.. J Clin Invest.

[PDF_00442] Bingel M., Arndt W., Schulze M., Floege J., Shaldon S., Koch K. M., Götze O. (1989). Comparative study of C5a plasma levels with different hemodialysis membranes using an enzyme-linked immunosorbent assay.. Nephron.

[PDF_00436] Chenoweth D. E., Cheung A. K., Henderson L. W. (1983). Anaphylatoxin formation during hemodialysis: effects of different dialyzer membranes.. Kidney Int.

[PDF_00399] Chenoweth D. E. (1984). Complement activation during hemodialysis: clinical observations, proposed mechanisms, and theoretical implications.. Artif Organs.

[PDF_00451] Chenoweth D. E., Hugli T. E. (1980). Human C5a and C5a analogs as probes of the neutrophil C5a receptor.. Mol Immunol.

[PDF_00476] Cohen M. S., Elliott D. M., Chaplinski T., Pike M. M., Niedel J. E. (1982). A defect in the oxidative metabolism of human polymorphonuclear leukocytes that remain in circulation early in hemodialysis.. Blood.

[PDF_00396] Craddock P. R., Fehr J., Dalmasso A. P., Brighan K. L., Jacob H. S. (1977). Hemodialysis leukopenia. Pulmonary vascular leukostasis resulting from complement activation by dialyzer cellophane membranes.. J Clin Invest.

[PDF_00463] Craddock P. R., Hammerschmidt D., White J. G., Dalmosso A. P., Jacob H. S. (1977). Complement (C5-a)-induced granulocyte aggregation in vitro. A possible mechanism of complement-mediated leukostasis and leukopenia.. J Clin Invest.

[PDF_00504] Fantozzi R., Brunelleschi S., Soldaini G. B., Blandina P., Masini E., Raimondi L., Mannaioni P. F. (1983). N-Formylmethionyl-leucyl-phenylalanine: Different releasing effects on human neutrophils and rat mast cells.. Agents Actions.

[PDF_00445] Freyria A. M., Leitienne P., Veysseyre C. N., Bringuier J. P., Traeger J. (1988). Complement C3 and C5 degradation products during hemodialysis treatment: study of an index of membrane bioincompatibility.. Int J Artif Organs.

[PDF_00422] Ganz T. (1987). Extracellular release of antimicrobial defensins by human polymorphonuclear leukocytes.. Infect Immun.

[PDF_00457] Goldstein I. M., Roos D., Kaplan H. B., Weissmann G. (1975). Complement and immunoglobulins stimulate superoxide production by human leukocytes independently of phagocytosis.. J Clin Invest.

[PDF_00460] Hammerschmidt D. E., Bowers T. K., Lammi-Keefe C. J., Jacob H. S., Craddock P. R. (1980). Granulocyte aggregometry: a sensitive technique for the detection of C5a and complement activation.. Blood.

[PDF_00490] Heierli C., Markert M., Lambert P. H., Kuwahara T., Wauters J. P. (1988). On the mechanisms of haemodialysis-induced neutropenia: a study with five new and re-used membranes.. Nephrol Dial Transplant.

[PDF_00424] Hugli T. E. (1981). The structural basis for anaphylatoxin and chemotactic functions of C3a, C4a, and C5a.. Crit Rev Immunol.

[PDF_00402] Hörl W. H., Feinstein E. I., Wanner C., Frischmuth N., Gösele A., Massry S. G. (1990). Plasma levels of main granulocyte components during hemodialysis. Comparison of new and reused dialyzers.. Am J Nephrol.

[PDF_00495] Hörl W. H., Riegel W., Schollmeyer P., Rautenberg W., Neumann S. (1986). Different complement and granulocyte activation in patients dialyzed with PMMA dialyzers.. Clin Nephrol.

[PDF_00501] Hörl W. H., Schaefer R. M., Heidland A. (1985). Effect of different dialyzers on proteinases and proteinase inhibitors during hemodialysis.. Am J Nephrol.

[PDF_00474] Klempner M. S., Gallin J. I., Balow J. E., Van Kammen D. P. (1980). The effect of hemodialysis and C5a des arg on neutrophil subpopulations.. Blood.

[PDF_00426] Klos A., Ihrig V., Messner M., Grabbe J., Bitter-Suermann D. (1988). Detection of native human complement components C3 and C5 and their primary activation peptides C3a and C5a (anaphylatoxic peptides) by ELISAs with monoclonal antibodies.. J Immunol Methods.

[PDF_00439] Knudsen F., Nielsen A. H., Pedersen J. O., Jersild C. (1985). On the kinetics of complement activation, leucopenia and granulocyte-elastase release induced by haemodialysis.. Scand J Clin Lab Invest.

[PDF_00404] Kolb G., Fischer W., Müller T., Höffken H., Joseph K., Lange H., Havemann K. (1989). Granulocyte-related bioincompatibility of hemodialysis: inhibition of oxidative metabolism, degranulation reaction, enzyme release and leukocyte sequestration in the lung.. Int J Artif Organs.

[PDF_00471] Kolb G., Höffken H., Müller T., Havemann K., Joseph K., Lange H. (1990). Kinetics of pulmonary leukocyte sequestration in man during hemodialysis with different membrane-types.. Int J Artif Organs.

[PDF_00420] MASSEY V. (1959). The microestimation of succinate and the extinction coefficient of cytochrome c.. Biochim Biophys Acta.

[PDF_00414] Maderazo E. G., Woronick C. L. (1978). Micropore filter assay of human granulocyte locomotion: problems and solutions.. Clin Immunol Immunopathol.

[PDF_00498] Maillet F., Kazatchkine M. D. (1991). Specific antibodies enhance alternative complement pathway activation by cuprophane.. Nephrol Dial Transplant.

[PDF_00481] Markert M., Heierli C., Kuwahara T., Frei J., Wauters J. P. (1988). Dialyzed polymorphonuclear neutrophil oxidative metabolism during dialysis: a comparative study with 5 new and reused membranes.. Clin Nephrol.

[PDF_00484] Nguyen A. T., Lethias C., Zingraff J., Herbelin A., Naret C., Descamps-Latscha B. (1985). Hemodialysis membrane-induced activation of phagocyte oxidative metabolism detected in vivo and in vitro within microamounts of whole blood.. Kidney Int.

[PDF_00453] Psychoyos S., Uziel-Fusi S. (1989). Comparison of LTB4- and C5a-stimulated chemotaxis of isolated human neutrophils: difference revealed by cell migration in thick filters using the multiwell cap procedure.. Agents Actions.

[PDF_00510] Schaefer R. M., Herfs N., Ormanns W., Hörl W. H., Heidland A. (1988). Change of elastase and cathepsin G content in polymorphonuclear leukocytes during hemodialysis.. Clin Nephrol.

[PDF_00479] Van Epps D. E., Garcia M. L. (1980). Enhancement of neutrophils function as a result of prior exposure to chemotactic factor.. J Clin Invest.

[PDF_00430] Webster R. O., Larsen G. L., Henson P. M. (1982). In vivo clearance and tissue distribution of C5a and C5a des arginine complement fragments in rabbits.. J Clin Invest.

[PDF_00433] Weisdorf D. J., Hammerschmidt D. E., Jacob H. S., Craddock P. R. (1981). Rapid in vivo clearance of C5ades arg: a possible protective mechanism against complement-mediated tissue injury.. J Lab Clin Med.

[PDF_00448] Yancey K. B., Lawley T. J., Dersookian M., Harvath L. (1989). Analysis of the interaction of human C5a and C5a des Arg with human monocytes and neutrophils: flow cytometric and chemotaxis studies.. J Invest Dermatol.

[PDF_00507] Zimmerli W., Reber A. M., Dahinden C. A. (1990). The role of formylpeptide receptors, C5a receptors, and cytosolic-free calcium in neutrophil priming.. J Infect Dis.

[PDF_00487] de Vinuesa S. G., Resano M., Luño J., Gonzalez C., Barril G., Junco E., Valderrabano F. (1983). Leucopenia, hypoxia and complement activation in haemodialysis. Three unrelated phenomena.. Proc Eur Dial Transplant Assoc.

